# A Pilot Study Comparing Aortic Valve Area Estimates Derived from Fick Cardiac Output with Estimates Based on Cheetah-NICOM Cardiac Output

**DOI:** 10.1038/s41598-020-64753-3

**Published:** 2020-05-12

**Authors:** Ludmil Mitrev, Noud van Helmond, Georges Kaddissi, Ahmed Awad, Kinjal Patel, Janah Aji, Jeffrey Ogbara, Zahi Rafeq, Vineeth Nagubandi, Debbie Orr, John Gaughan, Michael Rosenbloom

**Affiliations:** 1 Department of Anaesthesiology/Division of Cardiac Anaesthesia, Cooper University Hospital, One Cooper Plaza, Camden, NJ 08103 USA; 2 Department of Cardiology, Cooper University Hospital, One Cooper Plaza, Camden, NJ 08103 USA; 3 Department of Medicine, Cooper University Hospital, One Cooper Plaza, Camden, NJ 08103 USA; 4 Chief, Department of Cardiothoracic Surgery, Cooper University Hospital, One Cooper Plaza, Camden, NJ 08103 USA

**Keywords:** Cardiology, Interventional cardiology, Interventional cardiology

## Abstract

Cardiac output during cardiac catheterization is often estimated using the modified Fick method (CO_Fick_). In this proof-of-concept, prospective non-randomized study carried out in a single academic healthcare centre, we examined whether replacing CO_Fick_ in the Gorlin formula with Cheetah-NICOM monitor cardiac output (CO_Cheetah_) could produce an accurate and precise estimate of aortic valve area in patients with severe aortic stenosis. In twenty-six subjects, CO_Fick_ and CO_Cheetah_ were obtained concurrently. A spot and 3-minute running average of CO_Cheetah_ was used. Bland and Altman analysis was used to derive bias, 95% limits of agreement (LOA) and confidence intervals (CI). The mean difference (bias) between AVA_Cheetah_ (average) and AVA_Fick_ was 0.11 cm^2^ and the 95% LOA were ±0.42 cm^2^. The 95% CI of the bias was 0.02–0.2 cm^2^. The bias and 95% LOA of AVA_Cheetah_ (spot value) were 0.14 ± 0.42cm^2^, with a 95% CI of 0.06–0.23 cm^2^. No proportional bias was present. AVA_Cheetah_ thus appears to be a reasonably accurate measure of AVA in patients with severe aortic stenosis compared to AVA_Fick_ measured using a modified Fick CO. However, the limits of agreement were not narrow enough to consider AVA_Cheetah_ and AVA_Fick_ interchangeable.

## Introduction

Left and right heart catheterization is standard of care in the pre-operative evaluation of the patient with aortic stenosis (AS). Catheterization is performed to evaluate the coronaries and not simply to confirm the presence and degree of severe aortic stenosis. The decision to pursue intervention depends on many factors including the patient’s symptomatology, and is not based solely on the transvalvular gradient or the calculated stenotic orifice area.

Left heart catheterization assesses peak and mean pressure gradients across the aortic valve, the state of the patient’s coronary vasculature, and provides an angiographic assessment of left ventricular performance. Right heart catheterization is performed in order to measure the various pressures from the superior vena cava to pulmonary artery, to obtain mixed venous blood sampling and estimate cardiac output (CO) using the Fick method (CO_Fick_). In clinical practice, a modified Fick method is commonly used, which assumes oxygen consumption to be 125 millilitres x BSA (body surface area). Aortic Valve Area (AVA_Fick_) is then calculated using the Gorlin formula^1,2^.

We hypothesized that substituting CO derived from a Cheetah monitor (Cheetah Medical Inc., Newton Centre, Massachusetts, USA; CO_Cheetah_) in the Gorlin formula could produce accurate and precise estimates of AVA relative to AVA_Fick_. If this were the case, it could be possible to avoid right heart catheterization in patients in whom this procedure is performed solely for the purposes of obtaining CO_Fick_ via the modified Fick method. Using a non-invasive CO monitor in this fashion would not eliminate the assumptions of the Gorlin formula itself nor any potential errors deriving thereof.

## Results

After the subjects provided informed consent, we enrolled 26 subjects in the study in a consecutive, non-randomized fashion. Subjects were screened through the cardiac catheterization laboratory schedule. All patients in the final analysis had at least moderate AS. Only one subject had atrial fibrillation, but was permanently paced with an implanted pacemaker. All subjects had a tricuspid AV. There was one missing AVA_Echo_ value, which was not calculated during the subject’s TTE. In two patients the AV could not be crossed, and they were therefore unevaluable for the purposes of the study. In one subject, the aortic valve was found not to be stenotic, and that patient was also excluded from the Bland and Altman analysis. The final sample thus consisted of 23 subjects. No unexpected outliers or artefacts were encountered, and the patients were at steady state when the Fick and Cheetah measurements were recorded. This was verified via review of the Cheetah data export files and the catheterization procedure record. None of the subjects were noted to have cardiomegaly or dilatation of the aorta on their TTE reports or during aortography/ventriculography.

The baseline physiologic characteristics of the subjects are summarized in Table 1.

**Table 1:** Baseline physiologic characteristics of the subjects in the study sample.

	Age	Gender	Height (cm)	Weight (kg)	BSA (m^2^)	BMI (kg/m^2^)	Peak to peak AV gradient (mmHg)	Mean AV gradient (mmHg)
Mean	78.7	Male = 13 (57%)	166.2	87.8	1.9	30.4	41	43
Range	61–88		147–188	49–142	1.4–2.6	21–44	5–119	18–82

cm, centimetres; kg, kilograms; m^2^, meters squared; mmHg, millimetres mercury; BSA, body surface area; BMI, body mass index; AV, aortic valve.

Average and spot CO_Cheetah_ were substituted in the Gorlin formula (Eq. 2), deriving the corresponding average AVA_Cheetah_ and spot AVA_Cheetah_. The biases (mean differences), standard deviations, 95% limits of agreement, mean error percent and 95% confidence intervals are summarized in Table 2.

**Table 2:** Bias (mean difference), limits of agreement, confidence intervals and mean error percent (percent limits of agreement) for Cheetah CO and AVA, as well as echocardiogram-derived AVA, compared to the cardiac catheterization (Fick) reference values.

Variable	Mean Bias	Range of bias	SD	95% LOA	Mean error % (%LOA)	95%CI of bias	95%CI of LLOA	95%CI of ULOA
average CO_Cheetah_ − CO_Fick_ (L/min)	0.70	−1.89–3.35	1.28	±2.65	53%	0.15–1.25	−2.90– −0.99	2.39–4.30
spot CO_Cheetah_ − CO_Fick_ (L/min)	0.88	−2.06–2.95	1.24	±2.57	51%	0.35–1.42	−2.62– −0.76	2.52–4.38
average AVA_Cheetah_ − AVA_Fick_ (cm^2^)	0.11	−0.17–0.57	0.20	±0.42	51%	0.02–0.2	−0.46– −0.16	0.38–0.69
spot AVA_Cheetah_ − AVA_Fick_ (cm^2^)	0.14	−0.49–0.50	0.20	±0.42	51%	0.06–0.23	−0.42– −0.12	0.41–0.71
AVA_Echo_ − AVA_Fick_ (cm^2^)	−0.05	−1.25–0.91	0.37	±0.76	92%	−0.2–0.11	−1.08– −0.53	0.44–0.99

CO = cardiac output; SD = standard deviation; LOA = limits of agreement; Mean error % = %LOA = 95% limits of agreement expressed as percentage of the mean AVA_Fick_ or CO_Fick_, respectively; 95% CI = 95% confidence interval; LLOA = lower limit of agreement; ULOA = upper limit of agreement; average CO_Cheetah_ = CO_Cheetah_ averaged over the 3-minute period preceding the time of blood sampling for CO_Fick_; spot CO_Cheetah_ = CO_Cheetah_ obtained at the time of blood sampling for CO_Fick_ as a spot value; average AVA_Cheetah_ = AVA calculated with average CO_Cheetah_ in the Gorlin formula; spot AVA_Cheetah_ = AVA calculated with spot CO_Cheetah_ in the Gorlin formula.

**Figure 1:**
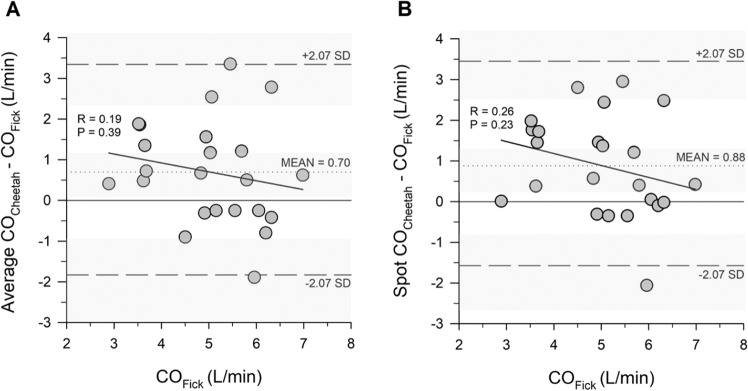
Bland and Altman plots showing the bias (mean difference) of average CO_Cheetah_ − CO_Fick_ (panel A) and spot CO_Cheetah_ − CO_Fick_ (panel B). The dashed lines show the 95% LOA defined as ± t_α, n−1_ *SD, where t_α_ is the t-value corresponding to n−1 degrees of freedom at an α error of 0.05, n is the sample size and SD = standard deviation. The sloped line represents a regression line quantifying the presence of proportional bias (if any). The lack of significant difference of the slope from 0 suggests lack of proportional bias. The Shapiro-Wilk test for normality was passed for regressions in the average (P = 0.64) and the spot data (P = 0.75). Shaded areas represent the 95% confidence intervals around the limits of agreement and the bias.

**Figure 2:**
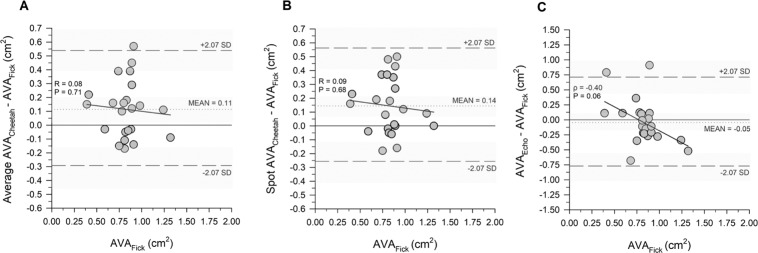
Bland and Altman plots showing the bias (mean difference) of average AVA_Cheetah_ (panel A), spot AVA_Cheetah_ (panel B), and AVA_Echo_ (panel C). The dashed lines show the 95% LOA defined as ± t_α, n−1_ *SD, where t_α_ is the t-value corresponding to n−1 degrees of freedom at an α error of 0.05, n is the sample size and SD = standard deviation. The sloped lines in panels A and B represent regression lines quantifying the presence of proportional bias (if any). The Shapiro-Wilk test for normality was passed for regressions in the average Cheetah (P = 0.19) and the spot Cheetah data (P = 0.45), but not in the AVA_Echo_ (P = 0.01). Therefore, a Spearman correlation line was plotted in panel C instead. The lack of significant difference of the slope from 0 suggests lack of proportional bias for the Cheetah or echo-derived AVA. Shaded areas represent the 95% confidence intervals around the limits of agreement and the bias.

The Bland and Altman plots for average CO_Cheetah_, spot CO_Cheetah_, average AVA_Cheetah_, spot AVA_Cheetah_, and AVA_Echo_ are presented in Figs. 1 and 2, along with regression lines and 95% CIs. CO_Fick_ and AVA_Fick_ were used on the x-axis per the methodology explained by Jan Krouwer^3^, as these were the measurements of reference.

**Figure 3:**
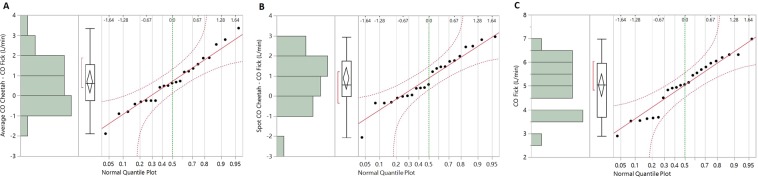
Normal quantile plots of the bias (mean difference) of average CO_Cheetah_ − CO_Fick_ (panel A), spot CO_Cheetah_ − CO_Fick_ (panel B), and CO_Fick_ (panel C). The plots indicate normal distribution of the data.

**Figure 4:**
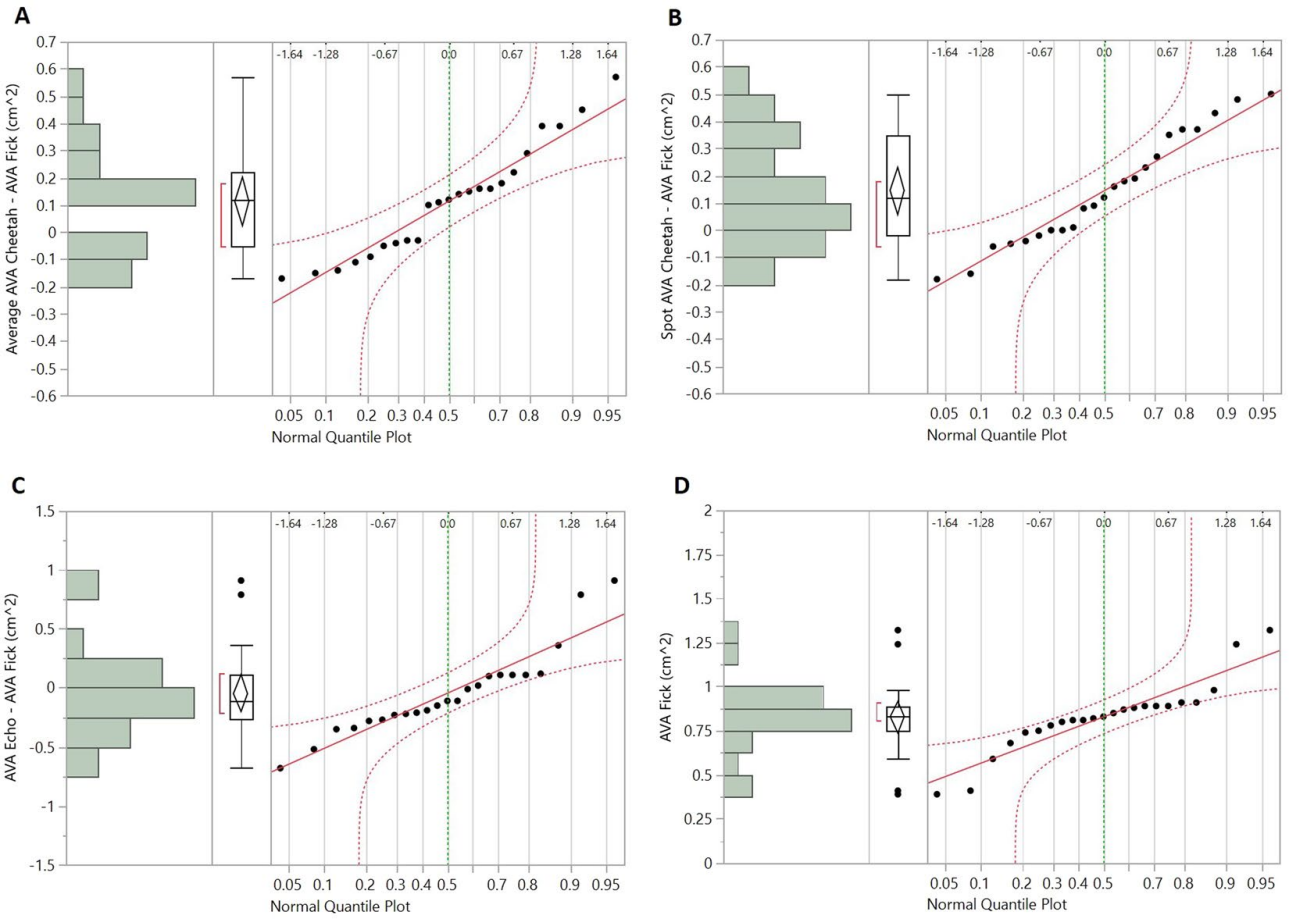
Normal quantile plots of the bias (mean difference) of average AVA_Cheetah_ (panel A), spot AVA_Cheetah_ (panel B) and AVA_Echo_ (panel C) from the reference method (AVA_Fick_). Panel D is a normal quantile plot for AVA_Fick_. The plots indicate normal distribution of the data.

The biases (mean differences) for CO and AVA were checked for normality using normal quantile plots as recommended by Montenij *et al*.^4^. The plots are shown in Figs. 3 and 4. The plots indicated normal distribution of the data.

**Figure 5:**
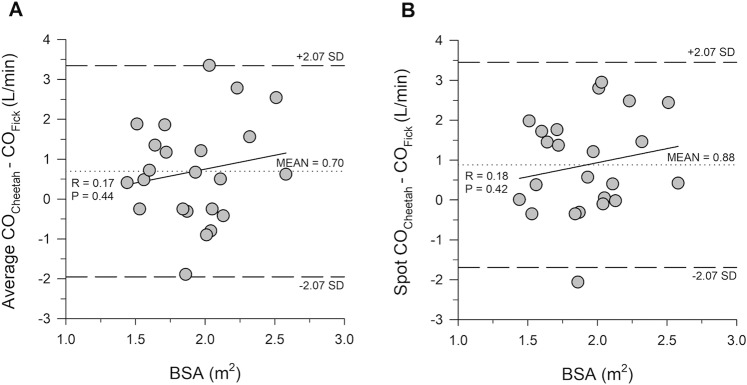
Average CO_Cheetah_ − CO_Fick_ (panel A) and spot CO_Cheetah_ − CO_Fick_ (panel B) plotted against BSA. The regression lines do not significantly differ from 0, indicating absence of bias proportional to BSA.

Figure 5 is a plot of the CO biases against BSA with a regression line used to demonstrate the absence of proportional bias. The lack of significant difference of the regression slope from 0 (p = 0.44 and p = 0.42 for average and spot CO_Cheetah_, respectively) indicates that there was no such bias related to BSA.

**Figure 6:**
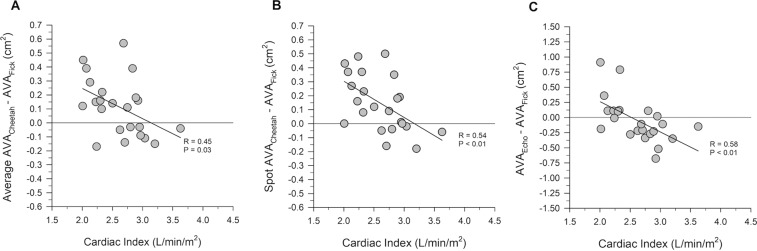
Linear regression analysis of average AVA_Cheetah_ −AVA_Fick_ (panel A), spot AVA_Cheetah_ − AVA_Fick_ (panel B), and AVA_Echo_ − AVA_Fick_ (panel C) vs. CI obtained from CO_Fick_. A normality test was passed by all the mean differences (biases).

We also plotted the biases of AVA_Cheetah_ and AVA_Echo_ against cardiac index obtained from CO_Fick_ and performed a linear regression analysis (Fig. 6). All regressions passed a normality test. We found a moderate correlation of the biases with CI (p < 0.05). This finding was expected, as both the Gorlin formula and the continuity equation are known to be flow-dependent^5^. The significant correlation between the biases and CI further indicates that there are different response characteristics between the Cheetah and Fick estimations of AVA with increases of CO. The constant between the two indicates a predictability between the two measurements with changes in CO, while not being interchangeable. Because CI also depends on BSA, we assessed if the difference between the AVA_Cheetah_ and AVA_Fick_ measurements was associated with BSA. We performed linear regression between BSA and the AVA_Cheetah_ − AVA_Fick_ measurements. We found that the difference between average AVA_Cheetah_ (R = 0.06, p = 0.78) or spot AVA_Cheetah_ (R = 0.09, p = 0.69) and AVA_Fick_ was not significantly associated with BSA.

## Discussion

The concept of using a non-invasive CO monitor to estimate AVA in patients undergoing cardiac catheterization for pre-operative evaluation of AS has, to our knowledge, not been studied in the past. Our main objective was to compare AVA calculated by substituting CO_Cheetah_ for CO_Fick_ in the Gorlin formula. If replacing the modified Fick CO with CO_Cheetah_ produced accurate and precise results over an acceptable range of AVA, the more invasive right heart catheterization could theoretically be eliminated in patients who do not have right sided pathology (e.g. shunts) that requires invasive evaluation. This could potentially increase overall patient safety and decrease risk, procedure time, complications and perhaps also cost. The use of the Cheetah-NICOM monitor would seem suitable during cardiac catheterization as it can be used in spontaneously-breathing patients and is completely non-invasive. For the estimation of AVA, a single reliable CO measurement would be required (perhaps averaged over several minutes). The use of CO_Cheetah_ would not eliminate the known shortcomings of the Gorlin formula. These include its assumption of a constant flow through a fixed orifice, its lack of accounting for the inertia to leaflet opening introduced by the diseased valve, the lack of accounting for valvular load and pulsatile arterial load, and the assumed quadratic relationship between blood flow and the pressure gradient^6,7^. Whether a continuous CO monitor used in this way accurately measures trends in CO over time, for which a variety of assessment methods have been proposed^4,8^, is immaterial in this scenario and was not tested.

We found the bias of AVA_Cheetah_ vs. AVA_Fick_ to be acceptable (0.11 and 0.14 cm^2^ for average and spot AVA_Cheetah_, respectively). No proportional bias was present, and the biases were normally distributed. However, the 95% LOA were wider than our desired clinical equivalence cut-off. In comparison to the Cheetah data, AVA_Echo_ had a smaller bias (−0.05 cm^2^), but even wider limits of agreement vs. AVA_Fick_. This discrepancy is hardly surprising given that AVA_Echo_ is a measurement relying on Doppler ultrasound and the continuity equation, whereas AVA_Cheetah_ relies on CO and the empirical relationships and assumptions inherent in the Gorlin formula. In addition, most of the patients in our sample had received small amounts of fentanyl and/or midazolam for comfort, whereas none had been sedated during their echocardiograms. This can be considered a limitation of our study. Nevertheless, it can be concluded that neither AVA_Cheetah_ nor AVA_Echo_ are clinically equivalent to AVA_Fick_ in this setting.

CO_Cheetah_ vs. CO_Fick_ merits some additional consideration. Non-invasive CO monitors have variable biases and limits of agreement relative to a method of reference such as the Fick or thermodilution CO (TDCO)^9–14^. Prior studies comparing Cheetah-NICOM to a reference method have shown smaller biases and LOA for CO_Cheetah_ than the ones we found in our study. Squara *et al*. compared the performance of the Cheetah-NICOM monitor to a continuous TDCO system in 110 intensive care patients, and found CO_Cheetah_ to have a bias of +0.16 L/min with LOA of ±1.04 L/min^15^. Another multi-institution study in intensive care patients (n = 70) demonstrated a bias of −0.09 L/min, with LOA ± 2.4 L/min, when the Cheetah monitor was compared to TDCO^16^. A study by Rich *et al*. compared CO_Cheetah_ to both CO_Fick_ and TDCO during right heart catheterization in 24 subjects with pulmonary hypertension^17^. In this study, bias and LOA of CO_Cheetah_ vs. CO_Fick_ were 0.21 ± 2.3 L/min. Bias and LOA of CO_Cheetah_ vs. TDCO were −0.37 ± 2.6 L/min. For comparison, bias and LOA of CO_Fick_ vs. TDCO were −0.91 ± 2.1 L/min.

We calculated the mean error % of CO_Cheetah_ and AVA_Cheetah_ as recommended by Montenij^9^, a value which has also been referred to as %LOA^18,19^. In order to determine whether a new method of CO measurement can be considered equivalent to a reference method, one has to take into account the precision of the reference method. An imprecise reference technique will lead to wide LOA and high mean error of measurement (ME) independent of the precision of the new method (in this case, CO_Cheetah_), because:

1 $$ME=\sqrt{{(experimentalprecision)}^{2}+{(referenceprecision)}^{2}}$$

For^4,18^ the methods to be interchangeable, the experimental precision should not exceed the reference precision.

Few reports exist in the literature providing data on the precision of CO_Fick_ as compared to another reference method such as TDCO. From the data in the aforementioned report by Rich *et al*.^17^, we were able to calculate the precision of CO_Fick_ as 44%. A study by Dhingra *et al*. in critically ill patients revealed an overall precision of CO_Fick_ of 64% across a wide range of CO values^9^. Conservatively accepting the lower of these values as the lowest acceptable precision of an experimental method and substituting this value (0.44) in Eq. 1, we obtain a ME of 63%. In other words, the mean error percent of the test method should not exceed this value in order to be considered of equivalent (interchangeable) precision as CO_Fick_. In our study, the mean error of CO_Cheetah_ (3-minute average) was 53%, so it would appear to fit this criterion. However, our absolute bias of CO_Cheetah_ was higher compared to that found in the aformentioned studies^9,15–17^. We wish to once again stress that our study should not be perceived as a true CO monitor validation study. For this to have been the case, we would have measured O_2_ consumption in the calculation of CO_Fick_ and not used the estimated O_2_ consumption. Alternatively, in a CO validation study, TDCO could have been used as the reference method. In this study, our main goal was to compare AVA_Cheetah_ to AVA_Fick_, with the latter value being calculated using estimated O_2_ consumption. The rationale was to examine the possibility of eliminating right heart catheterization where it is not otherwise indicated, and replace it with a completely non-invasive CO monitor.

A limitation of this study was the relatively small sample size. We assessed the agreement between the Cheetah and the Fick measurements using Bland-Altman analysis. Bland-Altman analysis is not a statistical test and performing power analysis is thus challenging^4^. Before the study commenced, we defined the systematic bias (0.1 cm^2^) and limits of agreement (±0.2 cm^2^) that we deemed acceptable for the primary outcome AVA measurement to conclude that the Cheetah and Fick AVA measurement would be interchangeable. We found that the systematic bias was close to the predefined threshold (0.11 and 0.14 cm^2^, for average and spot Cheetah values), whereas limits of agreement were considerably wider than the predefined threshold (±0.42 cm^2^ and ±0.42 cm^2^, Table 2 and Fig. 2). This let us to conclude that the two methods are not interchangeable, since one predefined criterion was not met. To assess the influence of the sample size on the findings we calculated 95% confidence intervals around the lower and upper limits of agreement^20^. We found that upper and lower boundaries of these respective limits were still beyond the predefined threshold (±0.27 and ±0.27, Table 2 last two columns). These results indicate that it is unlikely that including more subjects would have led to a different conclusion for our primary outcome (i.e. that we would have concluded that the Cheetah AVA estimation is interchangeable with Fick AVA estimation at the predefined threshold for limits of agreement).

In conclusion, based on this pilot study, we cannot recommend that right heart catheterization and the modified Fick CO be replaced by Cheetah-NICOM CO in the evaluation of the stenotic aortic orifice area. The bias of AVA_Cheetah_ was 0.11 and 0.14 cm^2^ (average and spot values, respectively), which is a slight overestimation of orifice area and would likely not lead to the patient being turned down for a necessary intervention if their aortic valve were sufficiently stenotic. However, the 95% LOA were ±0.42 cm^2^. We consider this too wide to claim that AVA_Cheetah_ is interchangeable with AVA_Fick_. The value this pilot study brings to science is that it highlights the potential role a non-invasive CO monitor could play in the evaluation of AVA. More studies could further refine this concept.

## Methods

### Cheetah non-invasive cardiac output monitor

The Cheetah-NICOM monitor uses bioreactance to measure cardiac output in a non-invasive fashion^15,16,21^. Bioreactance is based on the phase shifts of an alternating current applied to the thorax, produced by the pulsatile flow of blood in the large thoracic arteries. These phase shifts between the applied alternating current and the measured thoracic voltage are tightly correlated with the stroke volume (SV). By accurately and continuously measuring phase shifts, the monitor determines SV using 4 electrodes applied to the chest wall, two above and two below the diaphragm on each side of the thorax^12^. The NICOM signal effectively measures the blood volume change in the thorax between systole and diastole. The measurement can be performed both in spontaneously breathing and mechanically ventilated patients, and in patients with arrhythmias. Unlike bioimpedance-based CO monitors, the bioreactance Cheetah method has the advantage of being independent from the distance between the electrodes^11^. Because the Cheetah monitor electrodes are paired (one set on either side of the body), two separate CO signals are obtained and then averaged to produce the final CO measurement. The Cheetah CO values were exported as 1-minute running averages from the Cheetah monitor for the duration of the procedure.

### Inclusion and exclusion criteria

Subjects were enrolled if they met the following criteria: (i) were at least 18years of age, (ii) were able to provide signed written informed consent to participate in the study, (iii) had a diagnosis of at least moderate aortic stenosis (AS) on transthoracic echo (TTE), and (iv) the subjects’ height and weight could be accurately obtained prior to entering the study.

Subjects were excluded if the following conditions were present: (a) aortic or tricuspid valve regurgitation; (b) atrial fibrillation with irregular rhythm (i.e., not paced); (c) intra-cardiac shunt; (d) the use of intra-aortic balloon pump; (e) intubated or unconscious patients; (g) patient known to be pregnant; (h) emergency heart catheterization; (i) uncompensated congestive heart failure; (j) suspected significant hypovolemia; and (k) subject currently participating in an investigational drug or device study that interferes with the study endpoints.

### Catheterization laboratory procedures

The subjects underwent right and left heart catheterization. All time-stamped catheterization data, including CO_Fick_, peak and mean transvalvular gradients, and the systolic ejection period (SEP) were obtained using the McKesson Cardiology Station Release 13.0 (McKesson Corporation, San Francisco, CA). The study subjects received an average of 41 micrograms of fentanyl (range: 0–100; two did not receive any fentanyl) and 0.96 milligrams of midazolam (range: 0–2; five subjects did not receive any midazolam). CO_Fick_ was obtained from mixed venous and aortic blood samples. CO_Cheetah_ was obtained by applying the Cheetah monitor electrodes to the chest wall for the duration of the catheterization. Two sets of CO_Cheetah_ values were derived: the spot value from the Cheetah monitor at the time of CO_Fick_ (spot CO_Cheetah_), and a 3-minute average (average CO_Cheetah_). These were derived from the data export file of each case provided by the Cheetah monitor. The monitor clock was synchronized to that of the McKesson system. The Cheetah export file lists CO as minute-by-minute values. The spot value was the one listed at the time of blood sampling for CO_Fick_, and the average CO_Cheetah_ was the average of the two preceding minute values and the spot value (a 3 minute consecutive average). CO_Fick_ and AVA_Fick_ were considered the methods of reference for the purposes of this study. AVA_Echo_ was obtained from the subjects’ referral echocardiograms. TTE-derived AVA (AVA_Echo_) was calculated using the continuity equation from 2D images. None of the patients had 3D TTE, or any form of transoesophageal echocardiograms. No subject had been given any sedative during their echocardiography exam.

Calculations

2 $${\rm{Gorlin}}\,{\rm{Formula}}:AVA=\frac{{\rm{CO}}/{\rm{SEP}}}{44.3\,\times \sqrt{{\rm{Mean}}\,{\rm{Pressure}}\,{\rm{Gradient}}}}$$

where SEP = Systolic Ejection Period in seconds per minute obtained at the time of the crossing of the aortic valve; the empiric constant in the formula for a tricuspid aortic valve is 44.3, and for a bicuspid valve, 37.7; CO = cardiac output in liters/minute; AVA = aortic valve area.

3 $${\rm{Fick}}\,{\rm{Equation}}:CO=\frac{{{\rm{VO}}}_{2}}{({{\rm{CaO}}}_{2}-{{\rm{CvO}}}_{2})\times 10}$$

where CO = cardiac output, VO_2_ = O_2_ consumption, CaO_2_ = arterial oxygen content, CvO_2_ = venous oxygen content. VO_2_ was estimated per cardiac catheterization laboratory standard operating procedure as 125 millilitres O_2_ × Body Surface Area (BSA), CaO_2_ = 1.36 × Hgb [mg/dl] × SaO_2_, CvO_2_ = 1.36 × Hgb [mg/dl] × SvO_2_. SaO_2_ = arterial oxygen saturation, SvO_2_ = mixed venous oxygen saturation (from proximal pulmonary artery), Hgb = haemoglobin.

#### Statistical methods

Accuracy of AVA_Cheetah_ and CO_Cheetah_ was assessed using Bland and Altman analysis for bias and 95% LOA^4,20^. No power calculation was performed because the study was exploratory, the Bland and Altman method is descriptive and is not a statistical test, and most importantly, there was no historical data for AVA measured with the aid of a Cheetah monitor. To set the bias and LOA of AVA_Cheetah_ in perspective, these parameters were also calculated for AVA_Echo_. Limits of agreement are presented as ±t_α, n−1_*SD, where t_α_ is the t-value corresponding to n−1 degrees of freedom at an α error of 0.05, n is the sample size and SD = standard deviation. Regression was used to check for the presence of proportional bias. This was done not only for the mean differences (biases) between AVA_Cheetah_, AVA_Echo_, CO_Cheetah_ and their respective Fick-derived comparators, but also for the biases of CO_Cheetah_ against BSA, because BSA is the sole other variable in the formula for estimated VO_2_. Normal quantile plots of the bias (mean difference) of CO and AVA were used to visually verify that the biases were normally distributed. The mean error percent (percent LOA) was derived as the LOA divided by the mean AVA_Fick_ or mean CO_Fick_, respectively. Finally, 95% confidence intervals (CI) were calculated using the formulas proposed in the original Bland and Altman paper:

4 $$95 \% \,{\rm{CI}}\,{\rm{of}}\,{\rm{the}}\,{\rm{bias}}={\rm{bias}}\pm {t}_{\alpha ,n-1}\ast \sqrt{\frac{S{D}^{2}}{n}}$$

5 $$95 \% \,{\rm{CI}}\,{\rm{of}}\,{\rm{the}}\,{\rm{LOA}}=({\rm{upper}}\,{\rm{or}}\,{\rm{lower}}){\rm{LOA}}\pm {t}_{\alpha ,n-1}\ast \sqrt{\frac{3\ast S{D}^{2}}{n}}$$

where t_α_ is the t-value corresponding to n−1 degrees of freedom at an α error of 0.05, n is the sample size and SD = standard deviation.

Equivalence between the methods of calculating aortic valve area would be present if the mean bias (difference) was on the order of 0.1 cm^2^, since such a difference would unlikely have an undesirable diagnostic implication (i.e., underestimating the degree of stenosis and deferring intervention). For the same reason, the limits of agreement and their corresponding 95% confidence intervals would have to be narrow. No consensus as to how narrow exists, since replacing right heart catheterization with a non-invasive cardiac output measurement has not been attempted in a clinical setting before. We found it reasonable to accept absolute limits of agreement of no more than 0.2 cm^2^ if the methods were to be accepted as clinically equivalent.

The Institutional Review Board (IRB) of our hospital approved the study (IRB #16–004EX). All procedures performed in this study were in accordance with the ethical standards of the local IRB and with the 1964 Helsinki declaration and its later amendments or comparable ethical standards. Informed consent was obtained from all individual participants included in the study.
